# Emerging Trends in Biological Treatment of Wastewater From Unconventional Oil and Gas Extraction

**DOI:** 10.3389/fmicb.2020.569019

**Published:** 2020-09-09

**Authors:** Shwetha M. Acharya, Romy Chakraborty, Susannah G. Tringe

**Affiliations:** ^1^U.S. Department of Energy Joint Genome Institute, Lawrence Berkeley National Laboratory, Berkeley, CA, United States; ^2^Department of Ecology, Earth and Environmental Sciences Area, Lawrence Berkeley National Laboratory, Berkeley, CA, United States; ^3^Environmental Genomics and Systems Biology, Lawrence Berkeley National Laboratory, Berkeley, CA, United States

**Keywords:** hydraulic fracturing, produced water, reuse, biological treatment, metagenomics

## Abstract

Unconventional oil and gas exploration generates an enormous quantity of wastewater, commonly referred to as flowback and produced water (FPW). Limited freshwater resources and stringent disposal regulations have provided impetus for FPW reuse. Organic and inorganic compounds released from the shale/brine formation, microbial activity, and residual chemicals added during hydraulic fracturing bestow a unique as well as temporally varying chemical composition to this wastewater. Studies indicate that many of the compounds found in FPW are amenable to biological degradation, indicating biological treatment may be a viable option for FPW processing and reuse. This review discusses commonly characterized contaminants and current knowledge on their biodegradability, including the enzymes and organisms involved. Further, a perspective on recent novel hybrid biological treatments and application of knowledge gained from omics studies in improving these treatments is explored.

## Introduction

Technological advances have led to unprecedented growth in unconventional oil and gas extraction in the US over the last few decades (U.S. Energy Information Administration [EIA], 2020). This encompasses extraction of oil and/or natural gas from a wide variety of formations (e.g., shale, tight sands, and coal deposits/seams) through hydraulic fracturing (HF, also called “fracking”). HF involves introducing water mixed with other additives (collectively called Fracturing Fluid) under high pressure to open and propagate fractures in the target formation. Release of oil or gas from the formation is often accompanied by large quantities of wastewater. During the early production phase, this wastewater mainly consists of injected fluid mixed with formation brine, often called fracturing flowback (FFB). As production progresses, the proportion of formation brine increases and the wastewater is referred to as Produced water (PW). In 2017 alone, PW volume from major US shale plays was estimated to be about 600 billion liters (Scanlon et al., 2020). In this review, we discuss the potential applications of biological treatment to this wastewater, focusing mainly on wastewater generated from HF of shale oil and gas. Wastewaters generated during various stages are hereafter collectively referred to as Flowback and Produced water (FPW).

Current disposal methods mainly rely on reinjecting FPW into newly drilled wells or disposal into deep Underground Injection wells. Disposal into underground injection wells irreversibly removes water from the hydrological cycle and has raised concerns due to induced seismicity (Walsh and Zoback, 2015; Langenbruch and Zoback, 2016). Alternative disposal practices include on-site evaporation or seepage pits which have the potential to contaminate surface and groundwater (Vengosh et al., 2014; Chittick and Srebotnjak, 2017). Beneficial reuse has been very minimal, mainly for agricultural and landscape irrigation in California and Wyoming (Chittick and Srebotnjak, 2017; Ground Water Protection Council [GWPC], 2019; U.S. EPA, 2019). With increasing water stress and reuse of FPW gaining momentum, the US Environmental Protection Agency (EPA) is conducting studies to evaluate the management of oil and gas extraction wastewater generated at onshore facilities (U.S. EPA, 2019).

Among the 1,198 chemical compounds identified in FPW, only 14% have existing toxicity data and 24% can be detected through standard analytical methods (Danforth et al., 2020). Presence of such a wide range of identified and unidentified contaminants make FPW technologically challenging to treat. While physical technologies like desalination are attractive to treat FPW, they are often energy intensive and suffer from membrane fouling challenges (Butkovskyi et al., 2017; Chang et al., 2019). Thus, hybrid systems – which utilize biological treatment to reduce fouling upstream of physicochemical techniques that remove suspended as well as dissolved solids – are being studied (Abdollahzadeh et al., 2013; Riley et al., 2016; Butkovskyi et al., 2018; Kose Mutlu et al., 2018).

**FIGURE 1:**
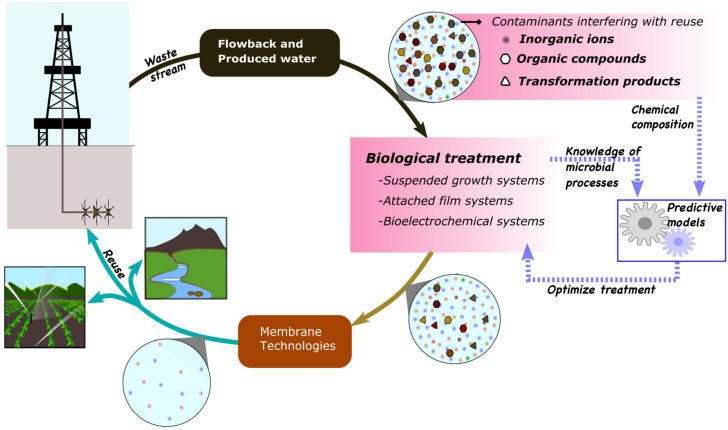
Improvements in biological treatment of flowback and produced water will require thorough understanding of contaminants and microbial processes involved.

This review aims to summarize recent trends in biological treatment of FPW with an emphasis on organic contaminants and microbial groups involved in their degradation (Figure 1). We briefly describe compounds found in FPW, including what is known about their degradation and how they may affect biological treatment and reuse of FPW, types of hybrid treatment modules developed, and microbial communities involved in remediation. Further, we explore the use of state-of-the-art metagenomics, transcriptomics, and proteomics along with metabolomics to improve understanding and optimization of biological treatment of FPW.

## Compounds of Concern

In order to effectively treat and reuse unconventional oil and gas wastewaters, it is essential to characterize contaminants that are present before and after treatment. High salt concentrations, which are common in FPW, can hamper quantification of both organic and inorganic constituents, rendering even baseline characterization challenging. Particularly, attenuation of gamma ray signals from radium during radioactivity measurements (Ajemigbitse et al., 2019), polyatomic interferences by chloride ion with carrier gas argon on arsenic quantification by inductively coupled plasma mass spectrometry (Balaba and Smart, 2012; Yaffe, 2014; Bolea-Fernandez et al., 2015) and formation of sodium adducts during analysis of organic compounds by mass spectrometry (Ferrer and Thurman, 2015; Oetjen et al., 2017; Nell and Helbling, 2019) are some of the major quantification issues reported in literature due to high salt concentration found in FPW. While methods for detecting hydrocarbons, especially the EPA priority pollutants^1^, are fairly well-documented and generally involve gas chromatography paired with mass spectrometry (GC-MS), the methods developed for surface and groundwater have to be modified to account for the complex matrix in FPW (Luek and Gonsior, 2017; Oetjen et al., 2017). FracFocus^2^, the chemical disclosure registry for hydraulic fracturing, provides information on the composition of fracking fluids injected into new wells, however, the amount of these HF chemicals persisting in the wastewater is not always known. Efforts are underway to characterize more polar, non-volatile components (e.g., HF chemicals like biocides, surfactants, etc.) using non-targeted Liquid Chromatography-Electrospray Ionization-Mass spectrometry (LC-ESI-MS) approaches, many of which provide semi-quantitative estimation of identified compounds (Ferrer and Thurman, 2015; Oetjen et al., 2018b; Nell and Helbling, 2019; Sun et al., 2019). An excellent description and comparison of physico-chemical characteristics of FPW across different shale regions can be found elsewhere (Chang et al., 2019). Moreover, several published reviews have described different aspects of ecotoxicity and health concern of various chemicals present in fracturing fluids, flowback and produced water (Stringfellow et al., 2014; Yost et al., 2016; Elliott et al., 2017; Folkerts et al., 2020) and will not be reviewed here. This section outlines various components of FPW that pose obstacles to reuse. Such compounds can be geogenic (from the formation), anthropogenic (HF additives) or transformation products of the two and are summarized in Table 1.

**TABLE 1:** Summary of major compounds present in FPW along with their biodegradability and toxicity concerns.

**Chemical compounds**	**Biodegradability^a^**	**Toxicity and other concerns**	**References**
*Geogenic compounds* Hydrocarbons and organic compounds			
VOCs (BTEX)	++	Reproductive toxicity; Neurotoxic; Carcinogen; soluble in water	Lueders, 2017; Varjani, 2017; Folkerts et al., 2020
PAHs (Naphthalene, Phenanthrene)	++	Genotoxic; Reproductive toxicity	Ghosal et al., 2016; Butkovskyi et al., 2017; Folkerts et al., 2020
Salts, radionuclides and other ions			
Major cations and anions (Na^+^, Ca^2+^, Mg^2+^, K^+^, Cl^–^, HCO${}_{3}^{-}$, SO${}_{4}^{2-}$, Br^–^)	−	Inhibit microbial degradation; Toxic to freshwater ecosystems; Disruption of soil structure and damage irrigational crops; Formation of disinfection byproducts in downstream drinking water plants	Oetjen et al., 2018a; Tasker et al., 2018; Sedlacko et al., 2019; Folkerts et al., 2020
Trace metal (Li, Ba, Fe, Mn, Sr, As, B, Pb)	−	Display toxicity based on concentration and bioavailability	Folkerts et al., 2020
NORM (Ra, U)	−	Carcinogen	Tasker et al., 2018
Fracturing fluid additives/Anthropogenic compounds			
Biocides (GA, DBNPA, QACs)	– to ++	Inhibit microbial degradation of co-contaminants in FPW	Ertekin et al., 2016; Rogers et al., 2017; Mumford et al., 2018; Akyon et al., 2019
Surfactants (PEG, PPG, AEOs, NPEOs)	+ to ++	No adverse effects known	Heyob et al., 2017; Rogers et al., 2018; Hanson et al., 2019
*Transformation products*			
Halogenated organic compounds	NA	Potential recalcitrant and toxic compounds	Hoelzer et al., 2016; Luek et al., 2018

*^*a*^ “−” not amenable to biological treatment, “+” potentially biodegradable, “++” highly biodegradable, NA, not available.*

### Geogenic Compounds

#### Hydrocarbons and Organic Compounds

Thousands of organic compounds have been detected in FPW and can originate either geogenically or from HF additives (Cluff et al., 2014; Orem et al., 2014; Khan et al., 2016; Hoelzer et al., 2016; Rosenblum et al., 2017b). Several chemical parameters such as Total Organic Carbon (TOC; amount of organic carbon per unit volume of water), Dissolved Organic Carbon (DOC; amount of organic carbon per unit volume of 0.45-micron filtered water), Chemical Oxygen Demand (COD; amount of oxygen required to oxidize all organics per unit volume of water) and less frequently, Total Petroleum Hydrocarbons (TPH; measured as extracted amount of petroleum hydrocarbons per unit volume of water) have been employed to quantify the total organic compounds in FPW. Dominant extractable hydrocarbons in PW samples from Marcellus, New Albany and Permian shales were reported to be alkanes, polycyclic aromatic hydrocarbons (PAHs) and straight-chain alkanes respectively (Orem et al., 2014; Khan et al., 2016). Not only does the organic composition of FPW vary with formation, it also varies during the lifetime of each well; DOC decreases steadily during the transition from FFB to PW (Cluff et al., 2014; Rosenblum et al., 2017b). Reports from Denver-Julesburg Basin FPW revealed that the hydrophilic fraction (e.g., simple organic acids, guar gum) is a major contributor of DOC, followed by transphilic acids (with intermediate polarity) and hydrophobic acids (compounds like humic acid, phenols, and cresols) (Rosenblum et al., 2017b).

Volatile Organic Compounds (VOCs), especially Benzene/Toluene/Ethyl Benzene/Xylenes (BTEX), are the most commonly analyzed hydrocarbons in FPW with concentrations varying from a few ***μ*** g/L to several mg/L depending on the age of the well and target formation (Khan et al., 2016; Chittick and Srebotnjak, 2017; Luek and Gonsior, 2017; Rosenblum et al., 2017b; Folkerts et al., 2020). BTEX are regulated carcinogens and highly mobile in water, posing serious risks to surface and groundwater resources. PAHs like naphthalene and phenanthrene are also frequently detected in wastewaters across different shale formations (Orem et al., 2014; Khan et al., 2016; Regnery et al., 2016; Chittick and Srebotnjak, 2017). Microbial remediation of BTEX, PAHs and petroleum-based hydrocarbons has been extensively studied in different environments including contaminated soil and sediment, surface and groundwater, marine systems, and wastewater from the petroleum industry (Ghosal et al., 2016; Lueders, 2017; Varjani, 2017). However, to the best of our knowledge, there are no studies investigating the specific biodegradation of these compounds in FPW.

#### Salts, Radionuclides, and Other Ions

Salinity is the measure of all dissolved salts in a given solution and generally determined using indirect methods such as conductivity or density (Baird et al., 2017). In characterization of FPW, salinity is often determined as Total Dissolved Solids (TDS, the portion of solids that passes through a filter membrane of pore size 2 microns or smaller). FPW salts are primarily composed of sodium (Na^+^) as the dominant cation, with calcium (Ca^2+^), magnesium (Mg^2+^), and potassium (K^+^) as minor cations, and chloride (Cl^–^) as the major anion, along with small amounts of bicarbonate (HCO${}_{3}^{-}$) and sulfate (SO${}_{4}^{2-}$) (Guerra et al., 2011; Chang et al., 2019). Trace metals frequently reported are lithium (Li), barium (Ba), iron (Fe), manganese (Mn), strontium (Sr), arsenic (As), boron (B), and lead (Pb) (Rosenblum et al., 2017b; Chang et al., 2019). Though the geology of the formation largely dictates the salinity and ionic composition of FPW, the age of the well and extraction processes also often play a role.

Kondash et al. (2017) categorized the major shale plays in the US based on salinity of the brine – high (TDS > 200,000 mg/L; Marcellus and Bakken formations), medium (TDS 50,000–100,000 mg/L; Haynesville, Barnett) and low (TDS < 50,000 mg/L; Niobrara, California and Eagle Ford). However, the salinity of FPW produced varies during the lifetime of a well, increasing gradually from the flowback period to produced water (Kondash et al., 2017; Rosenblum et al., 2017a). While iodide and ammonium ions were found to be dictated by geology and not by the extraction process (conventional vs. HF) (Harkness et al., 2015), a recent study found Ca/Mg vs. Ca/Sr as an effective tool to distinguish between contamination from shale gas PW, conventional hydrocarbon wastewater and non-impacted waters since these ratios vary with strata temperature and depth of the well at which conventional and HF operate (Tisherman and Bain, 2019). Additionally, naturally occurring radioactive materials (NORM; mainly radium (Ra), uranium (U), and their decay products) are frequently detected in FPW (Nelson et al., 2014; Rosenblum et al., 2017a).

A meta-analysis of published data on biological treatment of oilfield wastewater found average COD removal in actual wastewaters to be about 74% when TDS was low (i.e., TDS < 50,000 mg/L) (Camarillo and Stringfellow, 2018). Increase in salinity/TDS beyond this threshold has been shown to inhibit microbial degradation of contaminants in synthetic and real FPW (Lester et al., 2014; Akyon et al., 2015; Kekacs et al., 2015; Kose Mutlu et al., 2018; Hanson et al., 2019). Using halophilic bacterial consortia as inocula helped overcome these inhibitions, allowing stable bioreactor performance over a wide range of salinities (Pendashteh et al., 2010; Abdollahzadeh et al., 2014). However, the halophilic microbial consortia used in these studies were minimally characterized.

Treatment of a complex matrix like FPW should not only meet the requirements of the intended application but also be cost-effective. TDS greater than 40,000 mg/L leads to elevated desalination costs, thus, low TDS western brines (Niobrara, California and Eagle Ford) are more likely to be treated and reused (Chittick and Srebotnjak, 2017; Kondash et al., 2017).

Experiments simulating a PW spill (Niobrara, CO) on soil concluded that the high salt content of PW can disrupt soil structure and mobilize metal ions like copper, lead, aluminum and manganese during subsequent rainfall events (Oetjen et al., 2018a). Studies on irrigation of wheat with diluted untreated PW not only found reduction in plant development and yield (Sedlacko et al., 2019), but interestingly, a decrease in resistance to pathogens (Miller et al., 2019). Results of these studies indicate that factors beyond the salinity of PW, specifically concentrations of boron (salts of boron are added as crosslinkers) and nature of organic carbon, may play a major role in crop health and therefore must also be considered while determining the level of PW treatment necessary prior to use for irrigation. Prior to discharge of treated PW into streams, concentrations of bromide, iodide and ammonium must also be considered. Ammonia can adversely affect aquatic species in the receiving streams and promote algal growth; bromide and iodide can form toxic byproducts during disinfection (chlorination) in water treatment plants located downstream (Harkness et al., 2015).

### Fracturing Fluid Additives/Anthropogenic Compounds

HF involves addition of several chemicals to water that regulate viscosity to perform various functions ranging from generating fissures to ensuring smooth flowback from the formation (Elsner and Hoelzer, 2016). Among additives used, biocides, quaternary ammonium compounds (QACs) and surfactants have all been identified as compounds of concern owing to limited data on their aquatic toxicity and environmental persistence (Stringfellow et al., 2017). Additionally, solvents like acetone, methanol, isopropanol, naphthalene, 1,2,4- Trimethylbenzene, diesel and other petroleum distillates are frequently added during drilling operations (Elsner and Hoelzer, 2016) which may resurface in FPW along with geogenic organic compounds described in section “Hydrocarbons and Organic Compounds.” While more than one-third of 155 organic compounds used in HF had prior data demonstrating biodegradability (Camarillo et al., 2016), co-contaminant interactions can influence biodegradability of individual compounds in PW and need further investigation (Kekacs et al., 2015; Mclaughlin et al., 2016; Rogers et al., 2017; Akyon et al., 2018, 2019).

#### Biocides

Biocides are added to prevent biofouling by sulfate-reducing bacteria during HF and to prevent growth of microbes that clog the pipelines during gas production (Elsner and Hoelzer, 2016). Biocides used in HF have been classified into two major groups, oxidizing and non-oxidizing biocides; non-oxidizing biocides are further classified into lytic and electrophilic biocides based on their mode of action (Kahrilas et al., 2015). Although glutaraldehyde (GA) is the most frequently added biocide (Elsner and Hoelzer, 2016), the choice of biocide greatly varies with the type of formation, type of fracking, and the operator.

GA, a non-oxidizing, electrophilic biocide, inactivates bacteria by crosslinking amines present on bacterial membrane proteins (Maillard, 2002; Bartlett and Kramer, 2011). Hypersaline environments like PW can induce osmotic stress responses in bacteria, increasing resistance to GA, rendering it less effective in controlling microbial growth in such environments (Vikram et al., 2014). GA self-polymerizes into dimers and trimers, and this polymerization could impact its biocidal properties and residual concentration in the PW by precipitating out of solution. The rate of such transformation is governed by temperature, pH and salinity in experiments mimicking deep subsurface conditions (Kahrilas et al., 2016). Further, biodegradation rates and impact of GA on aquatic microbial communities can vary based on previous exposure to HF fluids, as experimentally demonstrated using HF impacted microcosms vs. pristine microcosms (Campa et al., 2018). GA addition increased lag phase for degradation of other organic additives/co-contaminants (acetate, guar gum, ethylene glycol, ethanol and isopropanol added individually) when treated by microbial mats (Akyon et al., 2019). Rogers et al. (2017) showed that GA trimers also exhibit bactericidal properties and inhibit degradation of other compounds in HF fluid in the event of a spill.

DBNPA (2,2-dibromo-2-nitrilopropionamide) is the second most commonly used biocide (Elsner and Hoelzer, 2016), however little is known about its biological degradation. A recent study evaluated the effects of electrophilic biocides, DBNPA and bronopol (2-bromo-2-nitropropane-1-3-diol), on anaerobic communities using iron reduction as an indicator of microbial activity (Mumford et al., 2018). While both biocides caused initial inhibition of microbial activity, long-lasting inhibition of iron-reduction was seen in amendments with bronopol only in mesocosms not previously exposed to HF wastewater. This was primarily associated with loss of iron-reducing organisms in 16S rRNA based analysis. Microbial activity and community structure were less impacted by these biocides when the mesocosms were constructed from HF-impacted sediments. Thus, acclimation of inocula to biocides may reduce the lag time and enhance the rate of degradation of contaminants in PW.

Quaternary ammonium compounds (QACs or “quats”) are frequently used amphiphilic biocides that have been found to persist in FPW (Ferrer and Thurman, 2015; Oetjen et al., 2018b; Nell and Helbling, 2019; Sun et al., 2019). Benzalkonium chloride (BAC) and dodecyldimethyl ammonium chloride (DDAC), commonly disclosed compounds belonging to QACs, have been reported to be aerobically degraded by various species of *Pseudomonas* (Nishihara et al., 2000; Ertekin et al., 2016), *Thalassospira* sp. and *Bacillus niabensis* (Bassey and Grigson, 2011) isolated from diverse environments. However, the effects of QACs on biological degradation of other PW compounds have not been investigated.

#### Surfactants

Non-ionic surfactants, including both unsubstituted polyglycols [Polyethylene glycol (PEG) and Polypropylene Glycol (PPG)] and substituted ethoxylates [Alkyl ethoxylates (AEOs) and Nonylphenol ethoxylates (NPEOs)], are employed as emulsifiers, corrosion inhibitors and crosslinkers in HF. These surfactants are generally present as a mixture of homologs and are identified by calculation of Kendrick mass defect from LC-MS data (Thurman et al., 2014). A few recent studies have investigated biological attenuation of surfactants in the event of FPW spills (Heyob et al., 2017; Rogers et al., 2018) or in natural-gas wells during production (Evans et al., 2019) or during treatment for beneficial reuse (Hanson et al., 2019). Heyob et al. (2017) investigated removal of surfactants (Propylene glycol, PPG and commercial surfactants containing NPEOs, AEOs, and PPG-PEG co-block polymers) in soil-groundwater microcosms under anaerobic conditions. AEOs and NPEOs were removed completely whereas PPG attenuated slowly; and propanediol dehydratase gene cluster (*pduCDE*) from dominant Firmicutes was predicted to be responsible for surfactant degradation under anaerobic conditions. In another study on PEG and PPG degradation under aerobic conditions in sediment-groundwater microcosms amended with PW (Rogers et al., 2018), authors suggested that primary alcohol dehydrogenase (PA-DH) genes present in the dominant group *Pseudomonas* could explain the removal of PEG and PPG. Evans et al. (2019) demonstrated that PPG, PEG and AEOs were likely to be degraded by *pduCDE* followed by *pduP* (aldehyde dehydrogenase gene) mapped to *Halanaerobium* in PW samples and in laboratory experiments with an isolate, *Halanaerobium congolense WG10*.

### Transformation Products

A hydraulically fractured well contains several organic and inorganic compounds held at high pressure and temperature which can lead to degradation and/or formation of new compounds through both abiotic and microbial processes. Halogenated organic compounds were detected in FPW samples from Fayetteville Shale (Hoelzer et al., 2016), as well as from Marcellus Shale (Luek et al., 2018). No similar compounds were disclosed as additives, leading researchers to hypothesize that these compounds were transformation products formed *in situ*. Reaction with persulfate (added as a breaker in the fracturing fluid) and other abiotic processes were implicated in the production of these halogenated organic compounds, along with biotic transformation by iodide-oxidizing bacteria, specifically *Roseovarius* spp. (Luek et al., 2018). Formation of halogenated compounds of common fracking additives (cinnamaldehyde, epichlorohydrin and 2,2-dibromo-3-nitrilopropionamide) has been experimentally confirmed under simulated conditions of high pressure, high temperature, presence of oxidizing agents (ammonium persulfate) and salinity (Sumner and Plata, 2018). These halogenated compounds may need further monitoring as they are likely to be recalcitrant to biodegradation and pose health risks.

Therefore, treating the wide range of contaminants present in FPW requires creative, efficient solutions that incentivize reuse. While membrane technologies are the default choice for treating high-salinity wastewater, issues like membrane fouling and high energy consumption hamper their application to treat FPW (Butkovskyi et al., 2017; Chang et al., 2019). Biodegradability of the organic compounds present in FPW makes biological pretreatment a logical step in complementing membrane technologies. Types of biological reactors currently employed to treat FPW, challenges and opportunities with biological treatment are explored in the next section.

## Biological/Hybrid Treatment Systems

Several types of reactors, both bench-scale and lab-scale, have been evaluated for PW treatment in the past few years. Due to the limited number of studies performed on FPW generated from (or mimicking) HF operations, those based on oilfield PW with comparable TDS (generally produced during conventional extraction) have also been included. The reactor configurations and dominant microbial groups identified are summarized in Table 2.

**TABLE 2:** Different reactor configurations used to treat FPW and dominant microbial groups observed.

**Type of reactor**	**Reactor volume**	**Type of wastewater treated**	**TDS (mg/L)**	**Avg. COD removal (%)**	**Inoculum source**	**Dominant microbes in the reactor**	**Molecular method used**	**References**
AGS-SBR	Lab-scale: 3.6 L, H/D – 10	Synthetic FFB	12,500–50,000	79 (TOC)	Aeration tank of hypersaline WWTP	*Cellvibrionaceae, Rhodocyclaceae*, *Rhodobacteraceae*, *Phyllobacteriaceae*	16S rRNA amplicon sequencing	Zhang et al., 2018a
AS	NA	Real PW (China)	5,500–16,000	72	AS from oilfield bioreactor	*Pseudoalteromonas, Marinomonas, Flavobacterium, Novosphingobium*	PCR-DGGE	Wang et al., 2015
MBR	Lab-scale: 5.1 L	Real PW (Turkey)	9,200–24,900	70	Lab-scale MBR treating PW	*Azoarcus* sp., *Thauera* sp., *Rhodobacteraceae*, *Porphyrobacter* sp.	PCR-DGGE	Kose Mutlu et al., 2018
MBR	Lab-scale: 5 L	Synthetic PW	64,400	83	Enriched halophilic consortia	*Marinobacter* spp., *Halomonas* spp., *Psychroflexus halocasei, Idiomarina loihiensis*	Isolate 16S rRNA sequencing	Abdollahzadeh et al., 2013
MM	6-well plates	Synthetic media and real PW (PA, United States)	PW(A): 182,700; PW(B): 18,400	NA	10% (v/v) PW + AS from municipal WWTP	PW (A1/2): *Halomonas, Idiomarina, Marinobacter Marinobacterium*; PW (B): *Rhodospirillaceae, Vibrio, Flavobacterium*	Metagenomics + 16S rRNA amplicon sequencing	Akyon et al., 2015
BAF	Lab-scale: D- 5 cm, H-76 cm	Real PW and FFB (CO, United States)	10,460–18,170	80	GAC from WTP	Aerated BAF: *Flavobacteria*, Gammaproteobacteria; Non-aerated BAF: *Anaerolineae, Clostridia, Deltaproteobacteria*	16S rRNA amplicon sequencing	Freedman et al., 2017
MFC	100 mL	Synthetic and real FFB (China)	19,000	72	Anaerobic digestor, mature MFC effluent	Anode-*Desulfuromonadales*, Anolyte- *Propionibacteriaceae, Sulfurovum, Rhodocyclaceae, Prolixibacteriaceae*	16S rRNA amplicon sequencing	Zhang et al., 2018b
MFC	125 mL	Synthetic and real PW (Iran)	65,000	95	Hypersaline anaerobic pond	*Desulfobacterales, Burkholderiales, Methylobacter, Methylotenera*	16S rRNA amplicon sequencing	Roustazadeh et al., 2017

*AGS, Aerobic Granular Sludge; SBR, Sequencing Batch Reactor; AS, Activated Sludge; MBR, Membrane Bioreactor; MM, Microbial Mats; BAF, Biologically Active Filter; MFC, Microbial Fuel Cells; PW, Produced water; FFB, Fracturing Flowback; H/D, Height to diameter ratio; NA, Not available; TDS, Total Dissolved Solids; COD, Chemical Oxygen Demand; TOC, Total Organic Carbon; GAC, Granular Activated Carbon; WWTP, Wastewater Treatment Plant; WTP, Water Treatment Plant; PCR-DGGE, Polymerase Chain Reaction Denaturing Gradient Gel Electrophoresis.*

### Suspended Growth Treatment Systems

#### Activated Sludge and Sequencing Batch Reactors

Activated sludge (AS) systems are the most popular form of biological reactor for wastewater treatment around the globe. A typical AS system consists of an aerated tank for mixing microbial biomass with wastewater, followed by a settling/sedimentation unit to separate biomass (sludge) from treated water. Such systems are operated in continuous flow conditions and a portion of the settled sludge is often recycled back into the aeration tank. A sequencing batch reactor (SBR) consists of a single tank to perform all the steps (reacting, settling, and decanting) of an activated sludge and hence is operated in a batch mode. Both these approaches have been used to treat FPW, and a few studies have investigated the microbes found in these systems.

Pendashteh et al. (2010) operated a lab-scale (5L) SBR to treat synthetic PW (with added crude oil and salts) and real PW (from a Malaysian oilfield, Petronas) on 24 h cycles (1 h feeding, 21 h reacting and 1 h settling and decanting). The reactor was inoculated with a halophilic consortium of microbes enriched from oil-contaminated saline soil. COD removal decreased from 93 to 63% when the TDS of synthetic PW was increased from 35,000 to 250,000 mg/L. When real PW (TDS: ∼16,500 mg/L, COD: 1,240 mg/L) was fed following adaptation to synthetic PW, a COD removal of 83% was achieved. *Pseudomonas*, *Ochrobactrum*, *Corynebacterium* and *Burkholderia* were major bacterial groups identified from the reactor sludge using a Biolog Microlog^TM^ System.

Wang et al. (2015) treated PW from two different Chinese oilfields sequentially using AS and reported effective (72%) COD removal maintained with a change in PW source but accompanied by shifts in microbial community. In another study, two bench-scale (3.6L) Aerobic Granular Sludge based SBRs were seeded with sludge from the aeration tank of a plant treating highly saline wastewater (Zhang et al., 2018a). The reactors were able to successfully remove varying loads of COD (800–2,300 mg/L) from synthetic FFB (containing polyacrylamide, isopropanol, guar gum, ethylene glycol and inorganic salts) and tolerate salinity change from 12,500 to 50,000 mg/L. The authors attribute the successful degradation to pre-adapted halophilic bacteria from the inoculum and increased production of extracellular polysaccharides (EPS) leading to stabilization of sludge. *Cellvibrionaceae, Rhodocyclaceae, Rhodobacteraceae*, and *Phyllobacteriaceae* were some dominant groups that were found to be enriched at a TDS of 50,000 mg/L.

#### Membrane Bioreactors

A typical membrane bioreactor (MBR) consists of an aerated tank containing sludge fed with wastewater and a submerged membrane module to filter out the treated effluent. In high salinity wastewater like PW where bacterial floc formation is disrupted, MBRs can overcome the floc settling requirement and retain the biomass in the reactor.

Abdollahzadeh et al. (2013) treated synthetic PW containing a mix of crude oil and salts (TDS: 64,400 mg/L, COD: 600–1,800 mg/L) using a lab-scale MBR system inoculated with a halophilic consortium enriched from oil-contaminated saline soil. The authors observed about 83% COD removal and minimal membrane fouling despite the decrease in average particle size distribution of the sludge during the course of operation.

A lab-scale MBR was acclimated to high salinity in phases using PW from different wells to investigate impact of salinity on the microbial community (Kose Mutlu et al., 2018). Upon increasing salinity from phase I (TDS: 9,200 mg/L, COD: 1881 mg/L) to Phase II (TDS: 19,000 mg/L, COD: 859 mg/L), an increase in mean diameter of floc and improved filtration were observed. However, on gradual increase in salinity of the feed in Phase III (TDS: 24,900 mg/L, COD: 622 mg/L), the floc structure was disrupted (significant decrease in mean diameter of floc, ascertained by microscopic observations) and free particles led to membrane fouling (as evidenced by an increase in transmembrane pressure). The effect of salinity was also reflected in the microbial ecology of the sludge where a shift in dominant species was observed. The role of factors other than salinity, including the different indigenous microbes found in the PW sources and variations in organic composition of PW fed into the reactor, in shaping the sludge community was not explored.

### Attached Film Systems

#### Microbial Mats

Emulating the naturally occurring stratified microbial consortia (or mats) often found in hypersaline environments, engineered microbial mats have been grown and evaluated for PW treatment in the laboratory. Akyon et al. (2015) grew microbial mats on grass silage (2.5 cm diameter spheres) submerged in rich nutrient Luria Bertani media amended with 50,000 mg/L TDS and inoculated with 10% (v/v) of PW mixed with activated sludge. These mats were first tested for their ability to degrade 2500 mg/L of either acetate or guar gum in synthetic media at different TDS levels. In both conditions, COD removal rate deteriorated at TDS of 100,000 mg/L and nearly slowed to a halt at 200,000 mg/L TDS. Further, the degradation kinetics of guar gum amended PW samples (Sample A with TDS 182,702 mg/L and Sample B with TDS 18,400 mg/L) and their one-half dilutions (referred to as Sample A1/2 and B1/2) were tested. Microbial mats could reduce COD of all samples except undiluted Sample A. 16S rRNA sequencing of microbial mats at the start and end of loading cycles revealed a significant increase in the *Idiomarina* genus and *Rhodospirillaceae* family in Sample A1/2 and B, respectively.

Biological treatability of PW samples from Utica and Bakken shale was also studied using microbial mats (Akyon et al., 2018). These samples varied in their organic and inorganic composition and were diluted to 50,000 and 100,000 mg/L TDS. DOC removal ranged from 1 to 87% (the Bakken sample showed essentially no degradation). Qualitative correlation of undiluted PW composition with biodegradability showed that first order biodegradation rate (DOC removal rate) could be positively correlated with higher relative presence of polymers like PEG, PPG, and NPEOs while negatively correlated with long-chain fatty acids and heteroatoms containing bromine, sulfur, iodine or chlorine. The microbial community involved in biodegradation was not analyzed and other factors affecting biodegradability, specifically inorganic ions, metals and biocides, were not measured in this study.

#### Biologically Active Filtration

Biologically active filters (BAF) employ microbial biofilms attached to a filter media (most commonly activated carbon) to adsorb and degrade organic compounds in wastewater along with removal of suspended solids. BAF successfully removed organic matter (75–85% DOC removal) from three different wastewaters (Piceance basin PW, and Denver-Julesburg basin PW and FFB) with varying salinity (12,600–31,200 mg/L TDS) and organic composition (DOC: 35.6–732 mg/L) as a pretreatment to ultrafiltration and nanofiltration (Riley et al., 2016). This BAF pretreatment reduced the propensity for membrane fouling in the downstream membrane processes. On further testing three different Granular Activated Carbon (GAC) media, it was revealed that a pre-existing biofilm helped efficient removal of organics (Riley et al., 2018). However, apart from electron microscopy imaging, characterization of microbial communities in the biofilm was not performed.

The scalability of BAF was tested using bench-scale and lab-scale columns fed with different wastewaters (Piceance basin PW, Denver-Julesburg basin PW and Denver-Julesburg basin FFB) under different operating conditions like aeration, pretreatment, operating temperature and empty bed contact time (Freedman et al., 2017). Aeration improved removal of organic matter, pretreatment was effective when aeration was not provided and BAF achieved close to 80% COD removal from PW in 72 h. Denver-Julesburg basin FFB had an order of magnitude higher COD (and DOC) than the PW, but the BAF was able to adapt to this change in feed and achieved similar COD removal at longer retention time (167 h). 16S rRNA gene-based microbial community analysis of filter media and produced water revealed differing dominant groups in aerated filters (*Flavobacteria* (predominantly genus *Fluviicola*) and *Gammaproteobacteria*) over non-aerated filters (*Deltaproteobacteria, Clostridia*, and *Anaerolineae*).

### Bioelectrochemical Systems

Bioelectrochemical systems (BES) can simultaneously achieve reduction in organic carbon and salinity, making them a promising technology, and an advancement over other biological systems to treat PW. BES harness the microbial capacity to degrade organic compounds anaerobically to generate electrons. These electrons can then be transported, via a pair of electrodes (anode to cathode), to terminal electron acceptors like oxygen or the system can be maintained anaerobic to produce hydrogen.

Several configurations of BES have been tested to treat PW. Microbial Fuel Cell (MFC) is the simplest design consisting of two electrodes separated by an ion-exchange membrane in which the wastewater to be treated is added to the anode. Microbial Desalination Cell (MDC) is a modification of MFC with a pair of ion exchange membranes between anode and cathode resulting in three chambers. Microbial Capacitive Desalination Cell (MCDC), like MDC, has three chambers but the middle chamber contains electrodes for Capacitive Deionization. These electrodes have a large surface area that adsorbs organic and inorganic ions under applied electrical potential (Forrestal et al., 2015; Monzon et al., 2017; Shrestha et al., 2018). On the other hand, Microbial Electrochemical Cell (MEC) requires external electricity to perform electrolysis with the aid of microbes and can generate hydrogen as a byproduct (Ghasemi Naraghi et al., 2015).

MCDC desalinated 18 times faster than MDC and 5 times faster in COD removal during treatment of Piceance basin PW (Forrestal et al., 2015). A liter-scale MCDC reactor successfully treated both PW and FFB from Piceance for 2 years (Forrestal et al., 2016). In another study (Shrestha et al., 2018), PW from Bakken Shale (ND, United States; higher salinity than Piceance Basin) was treated with MFC and MCDC, and while MFCs performed better than MCDC in removing COD, MCDC removed twice the amount of dissolved solids. The authors suggested use of halophiles to improve MCDC performance when treating hypersaline PW. While the above studies explored the use of BES for treatment of PW, the key microbes involved in the process were not investigated.

MFCs operated with Barnett Shale PW and subsequently with saline media due to limited availability of PW were found to be dominated by *Halanaerobium prevalens* and *Marinobacter hydrocarbonoclasticus*, both originating from the PW (Monzon et al., 2017). PW from Cheshmeh Khosh oilfield, Iran used for MEC based hydrogen production identified *Lysinibacillus macroides* as the major bacteria (Ghasemi Naraghi et al., 2015). When PW from the same oilfield was treated in a MFC, *Desulfobacterales, Burkholderiales, Methylobacter*, and *Methylotenera* were identified as predominant organisms on working anodes (Roustazadeh et al., 2017). Zhang et al. (2018b) designed a sulfur-cycle mediated MFC to simultaneously remove COD and Fe. While sulfur-oxidizing bacteria, *Desulfuromonadales* and *Sulfurovum* were presumed to be involved in the oxidation of sulfide in PW, other groups belonging to *Bacteroidetes, Firmicutes, Proteobacteria*, and *Chloroflexi* were thought to mediate removal of organics.

### Other Treatment Strategies

Researchers are now looking beyond traditional methods to treat FPW. *Alcanivorax borkumensis* SK2, a marine hydrocarbonoclastic bacterium, was able remove n-alkanes and other hydrocarbons in PW and produce neutral lipids (wax-like esters of commercial value) (Sudmalis et al., 2018). Hopkins et al. (2019) showed that an algal polyculture (mainly *Cyanobacterium aponinum* and *Parachlorella kessleri*) with halophilic bacteria could grow in PW over a wide range of salinities (TDS of 15,000–60,000 mg/L) and the harvested algal biomass was suitable for biodiesel production. He et al. (2019) tested a combination of 4 microbial consortia and 10 aquatic plants to treat FPW. They found the synergistic action of activated sludge and water dropwort (an aquatic plant) to be a promising candidate for nutrient removal and reduction in aquatic ecotoxicity.

### Challenges and Opportunities With Biological Treatment:

Though biological treatment of FPW is promising and can be initiated with a readily available inoculum (municipal wastewater sludge), most reactors need lengthy acclimation periods (in which the TDS is gradually increased) which may not be practical for a field-operating full-scale reactor. Reactor parameters such as temperature, mixing/oxygenation and retention time will affect both reactor performance and community composition. Faster, more effective strategies are needed to achieve a consortium that can both tolerate the varying inorganic and organic composition of FPW and efficiently remove chemically diverse contaminants. Biological processes also have the potential to create new potentially harmful contaminants, e.g., iodinated organic compounds detected in the effluent of a BAF attributed to the activity of iodide-oxidizing bacteria (Almaraz et al., 2020). Thus, it is critical to design biological treatment strategies that can identify and address potentially harmful biological transformations and changes in toxicity of treated effluent.

On a brighter note, exploring biological treatment of FPW also presents a great opportunity. Besides reduction of organic matter, biological treatment may help in recovery of rare elements and bioprospecting. Microbes cope with the stresses of heavy metal and radionuclide exposure by several mechanisms including biosorption, bioaccumulation, biotransformation and biomineralization (Bader et al., 2018; Kolhe et al., 2018). While adsorption and accumulation of heavy metals and radionuclides in biomass may create a problem for disposal, understanding and harnessing these processes can potentially help us design better and more sustainable technologies to recover different elements from the NaCl-rich FPW and brines. A good example for bioprospecting is a recent study where an iodide-oxidizing bacterium belonging to genus *Roseovarius* (isolated from natural brines) was shown to be capable of leaching gold from its ore in the presence of iodide, in a process more ecofriendly than traditional cyanide leaching (Khaing et al., 2019).

## Importance of Metagenomics, Transcriptomics, Proteomics and Metabolomics in Optimizing Wastewater Treatment

Most biological water treatment methods have been developed empirically, with relatively little knowledge of the organisms involved or their interdependencies. Modern molecular methods offer the opportunity to gain a molecular-level understanding of the biochemistry and microbiology of these systems. While something is known about the taxonomy of reactor communities (Table 2) this information is insufficient to understand the complex underlying network of biochemical interactions taking place during treatment. Additionally, little is known of the evolutionary history of these microbes, for instance whether they originate from FPW or the added inoculum, how they change during the acclimation phase, and what roles they play in degrading contaminants. A number of wastewater treatment systems have been subjected to metagenomic and/or metatranscriptomic analysis to address such fundamental questions.

Researchers at the University of Wisconsin, Madison have extensively studied microbial communities carrying out Enhanced Biological Phosphorus Removal (EBPR), particularly the dominant phosphate-accumulating organism *Candidatus* Accumulibacter phosphatis (CAP) (Martín et al., 2006; Camejo et al., 2019). In fact, one of the very first genomes reconstructed based on assembly of shotgun metagenome data (a Metagenome Assembled Genome, or MAG) was that of a CAP strain in a lab-scale EBPR reactor (Martín et al., 2006). That study revealed key details about phosphate transport and metabolism in CAP, enabling a reconstruction of its metabolic pathways and paving the way for follow-up studies on strain dynamics and gene expression in both lab-scale and commercial-scale reactors (He and Mcmahon, 2011; Oyserman et al., 2016).

Another application of metagenomics and metatranscriptomics to wastewater treatment involved studies of reactors degrading terephthalate, a plastics byproduct, where a preliminary multi-organism pathway postulated based on early metagenome sequencing efforts was later expanded upon with a complex network of cross-feeding relationships that highlights the multilayered nature of these systems (Lykidis et al., 2011; Nobu et al., 2015). Finally, metagenomics studies of a continuous-flow bioreactor treating thiocyanate revealed key organisms and pathways in thiocyanate breakdown and pointed to the likely importance of biofilm formation to reactor efficacy (Kantor et al., 2017).

Omics approaches are being used to study the microbial ecology of the subsurface, including shale reservoirs and the impact of HF on them. Initial studies, mainly using 16S rRNA gene based community analyses, by several groups showed that PW harbors low diversity, halotolerant bacteria and archaea, mainly dominated by *Halanaerobium* (Murali Mohan et al., 2013; Cluff et al., 2014; Mouser et al., 2016; Lipus et al., 2017). It is interesting to note that FFB, when oxygen is present and TDS is low, is often dominated by groups like *Marinobacter* and *Arcobacter*, and shifts to fermentative bacteria (mainly *Halanaerobium*) and methanogenic archaea during later PW, when anoxic conditions develop and TDS rises (Cluff et al., 2014; Mouser et al., 2016; Evans et al., 2018).

Researchers at the Marcellus Shale Energy and Environment Laboratory (MSEEL) have further shed light on microbes that reside in shale with the help of metagenomics and metabolite profiling combined with supporting laboratory experiments. Daly et al. (2016) performed metagenomics and metabolite profiling on samples collected from Marcellus and Utica wells over a period of 328 days. Interactions between six major halotolerant bacterial and archaeal members, namely *Halanaerobium, Halomonadaceae, Marinobacter, Methanohalophilus, Methanolobus*, *and Frackibacter* (a new genus currently known to reside only in shales), were described based on their genomes, which in turn explained the changes in key metabolites observed in the samples collected. Viral predation of these major groups was also implicated in community dynamics and nutrient cycling. Further research revealed the viral (phage) diversity in shales and phage-induced lysis of dominant *Halanaerobium* was shown to release intracellular metabolites that support microbes in such ecosystems (Daly et al., 2019). As a byproduct of these extensive studies, several key microbial groups – *Halanaerobium* (Booker et al., 2017a), *Frackibacter* (Booker et al., 2017b), *Marinobacter* (Evans et al., 2018; Tummings et al., 2018), *Methanohalophilus* (Borton et al., 2018), and *Arcobacter* (Evans et al., 2018; Panescu et al., 2018) – have been isolated, sequenced and their role in shale formations uncovered. These studies have not only shaped our understanding of shale microbial ecology but also how microbial processes could be involved in well operational issues like corrosion, souring and clogging.

On the other hand, there are few metagenomic studies on bioreactors optimized to treat FPW or even petroleum refinery wastewater. Molecular-level understanding of FPW treatment, however, is critical to successfully targeting multiple high-priority contaminants under a wide range of environmental conditions. Accurate, predictive models of FPW treatment bioreactors would dramatically accelerate optimization by replacing empirical manual parameter adjustments with rapid *in silico* tests. Thorough molecular characterization of bioreactor communities with varying performance combined with machine learning could identify biomarkers of successful treatment and potentially enable current monitoring strategies to be augmented or even replaced by molecular methods, allowing early warning of adverse events affecting reactor performance. Eventually, molecular methods will become a key part of a wastewater engineer’s toolkit.

## Conclusion

Treatment of FPW is challenging due to its complex chemical composition coupled with geographical and temporal variability. The low cost and effective treatment options offered by biological treatment systems in treating municipal wastewater have made them ubiquitous. Only in the past decade have we been able to understand core microbes and microbial processes behind treatment efficacy and resilience of these systems to shocks and overloads. Many studies have shown that FPW is amenable to biological treatment and different treatment modules to treat FPW have been discussed. Several studies indicate halophiles are effective for FPW treatment, but a systematic study to identify these halophiles during operation of a reactor and understand the key degradation pathways is not available. Omics guided study of core microbes should aid in constructing a core microbial community with broad metabolic potential to handle diverse contaminants and salinities as are common in FPW. Aided with optimized reactor design and robust microbial communities, a hybrid decentralized treatment module capable of handling fluctuations in composition as well as quantity of FPW could be a reality. Such a system could reduce the impacts of accidental release, promote a sustainable hydrological cycle and safeguard the environment.

## Author Contributions

SA wrote the manuscript with inputs and edits from ST and RC. All authors contributed to the article and approved the submitted version.

## Conflict of Interest

The authors declare that the research was conducted in the absence of any commercial or financial relationships that could be construed as a potential conflict of interest.

## Footnotes

1 https://www.epa.gov/sites/production/files/2015-09/documents/priority-pollutant-list-epa.pdf

2 https://www.fracfocus.org
